# Multidrug-resistant bacterial infections in the liver transplant setting

**DOI:** 10.1007/s13304-024-01903-6

**Published:** 2024-06-25

**Authors:** Alberto Ferrarese, Marco Senzolo, Lolita Sasset, Domenico Bassi, Umberto Cillo, Patrizia Burra

**Affiliations:** 1 Multivisceral Transplant Unit, Department of Surgery, Oncology and Gastroenterology, Padua University Hospital, Padua, Italy; 2 Gastroenterology, Department of Surgery, Oncology and Gastroenterology, Padua University Hospital, Padua, Italy; 3 Infectious Disease Unit, Padua University Hospital, Padua, Italy; 4 Hepato-Biliary-Pancreatic Surgery and Liver Transplantation Unit, Padua University Hospital, Padua, Italy

**Keywords:** Klebsiella Pneumoniae, Acute-on-chronic liver failure, Antibiotics, Sepsis

## Abstract

Bacterial infections pose a life-threatening complication in patients with decompensated liver cirrhosis and acute-on-chronic liver failure. An increasing prevalence of infections caused by multidrug-resistant organisms (MDROs) has been observed in these patients, significantly impacting prognosis. A growing body of evidence has identified the most common risk factors for such infections, enabling the development of preventive strategies and therapeutic interventions. MDRO infections may also occur after liver transplantation (most commonly in the early post-operative phase), affecting both graft and patient survival. This review provides an overview of MDRO infections before and after liver transplantation, discussing epidemiological aspects, risk factors, prevention strategies, and novel therapeutic approaches. Furthermore, it examines the implications of MDRO infections in the context of prioritizing liver transplantation for the most severe patients, such as those with acute-on-chronic liver failure.

## Introduction

The liver plays a central role in the regulation of immune defense, being actively involved in the inflammatory response against bacteria through a complex interplay with the intestinal barrier and the immune system. It is also programmed to maintain tolerance to weak pro-inflammatory stimuli, especially those coming from the gut via the portal blood [1].

Cirrhosis is a systemic disease affected by a significant impairment of the immune system, especially at later stages. This impairment, known as cirrhosis-associated immune dysfunction, is mainly characterized by two factors. First, chronic systemic inflammation is primarily driven by increased bacterial translocation from the gut, closely associated with disease severity. Second, profound immune deficiency characterized by acquired immune system paralysis and an exhausted immune response to pathogens [2].

From a clinical perspective, understanding the pathophysiology helps to explain the high prevalence of bacterial infection (BI) in cirrhosis and its clinical impact on the natural history of the disease. On one hand, the close relationship between cirrhosis-associated immune dysfunction and disease stage explains why the sickest patients (often hospitalized) are at the highest risk of developing clinically significant BI episodes. On the other hand, immune paralysis explains why these patients, once BI occurs, are at high risk of hepatic and extrahepatic dysfunction (e.g., acute-on-chronic liver failure development) and ultimately face high short-term mortality [3].

In recent decades, clinical care of cirrhosis has improved dramatically due to a better understanding of pathophysiology and disease stage, development of new drugs, and the worldwide implementation of liver transplant (LT) programs. Unfortunately, such progress has only partially impacted the modulation of immune dysfunction, and BI continues to be a detrimental clinical event in the natural history of decompensated cirrhosis.

Infections caused by multidrug-resistant organisms (MDRO) represent a major public health problem worldwide, also posing a significant challenge in Hepatology [4–6]. Indeed, hepatology wards, LT Units, and liver Intensive Care Units (ICU) are observing an increasing prevalence of such infections over time. Antibiotic pressure, multiple hospitalizations, repeated contacts with healthcare facilities, and the performance of many diagnostic and therapeutic invasive procedures may partly explain this rising prevalence, especially in end-stage liver disease patients [7–9]. In recent years, multiple risk factors for the development of MDRO infections occurring in the early post-transplant phase have also been identified. These include pre-transplant factors and post-operative surgical and medical complications [10, 11]. Additionally, the prognostically relevant role of such infections for graft and patient survival has been highlighted. The MDRO infection therefore represents a clinical challenge, with some questions still unresolved, both in the pre-transplant and post-operative phases. The aim of this narrative review is to provide an updated overview of MDRO infections in such a setting, with special attention to patients with end-stage liver disease in the pre-transplant phase, and to patients acquiring infection by MDRO strains in the immediate post-operative period.

## MDRO infection in end-stage liver disease

### Prevalence

The prevalence of MDRO infections in cirrhosis varies across studies, and several factors may explain this heterogeneity. First, different definitions have been applied over time and across datasets, with many not meeting commonly adopted criteria (e.g., resistance to at least one agent in three different antibiotic classes). Second, systemic infection and colonization have often been used interchangeably, without providing useful information regarding the need for targeted antibiotic therapy. Third, MDRO prevalence may vary according to differences in surveillance protocols and/or healthcare facilities. Lastly, many infections in cirrhosis turn out to be culture-negative (e.g., pneumonia, spontaneous bacterial peritonitis), thus the actual prevalence could be underestimated.

According to available literature, MDR strains are responsible for 20–30% of culture-positive BI, with heterogeneity in prevalence and predominant strains across geographical areas, also highlighting environmental factors that could influence the spread of such infections. For instance, extended-spectrum beta-lactamase enterobacterales (ESBLE) are frequently isolated across Europe, North America, and Asia, whereas methicillin-resistant Staphylococcus aureus (MRSA) and vancomycin-resistant enterococci (VRE) predominate in North America. A prospective, observational, multicenter study that included forty-six centers worldwide reported that MDR accounted for 34% of culture-positive infections [12]. Data from a European cohort of more than 450 patients showed that 29% of culture-positive infections were caused by resistant rods [13]. Another multicenter study in Italy reported a 27% prevalence of MDR infections in 395 patients, mainly due to gram-negative rods [14]. These results were consistent with data from a large cohort of 876 patients listed for LT between 2006 and 2014, where the rate of MDR-related BI was 24.2% [15]. The prevalence of MDR pathogens in the ICU setting is variable according to studies [16, 17], but it may rise up to 30–50% of culture-positive infections.

### Risk factors

Many risk factors for MDRO infection have been identified in ESLD patients, the majority of these being shared with other chronic conditions: repeated infection and/or hospitalizations, previous/recent exposure(s) to systemic antibiotics, long-term ICU or hospital stay, indwelling or central catheters, invasive procedures.

Among preventable factors, the role of prior antibiotic course(s) is worth mentioning. Antibiotic overuse is a major healthcare problem derived from many causes as environmental exposure, diffuse spread in agriculture and farming. However, it is interesting that in cirrhosis, since half of BI episodes are usually culture-negative, a targeted antibiotic therapy is not always feasible, therefore the odds of long-lasting empiric therapies are higher, with reduced use of de-escalating strategies.

Antibiotic prophylaxis has been advocated as a further risk factor for subsequent MDR infection, but this point remains debated. First, it should be mentioned that, according to Guidelines, patients with cirrhosis who require long-term antibiotic prophylaxis are a restricted subpopulation, namely those with low ascites fluid protein concentrations and those with prior episodes of SBP [18, 19]. Data on the association between norfloxacin prophylaxis and MDRO infection came from observational studies [20, 21], whereas more recent multicenter experiences did not confirm these findings [12, 22]. There is significant evidence that prophylaxis may enhance the risk of MDRO colonization, which is, in turn, another known risk factor for MDRO infection.

The association between MDRO colonization and further systemic infection from the same strain has been largely demonstrated in patients with and without cirrhosis. A recent study showed that ICU patients with cirrhosis had a higher MDRO colonization rate than patients without, confirming that such colonization increased by 7-folds the risk of being infected by the same strain. Notably, this risk was observed both for Gram -ve and Gram + ve strains, in two different Countries [17].

### Diagnosis

Currently, a single biomarker or a combination of many is not sufficiently accurate for diagnosing bacterial infection in ESLD. Therefore, diagnosis should be based on integrating clinical suspicion, biomarkers, radiological features, and microbiological samples. In cirrhotic patients, rapid diagnosis and initiation of appropriate empirical (or sometimes targeted) antibiotic therapy are crucial for patient survival. Diagnostic kits based on various technologies (such as MALDI-TOF MS and multiplex PCR) are increasingly available. These kits can identify the pathogen(s) and determine their antimicrobial susceptibility within 1 to 6 h, facilitating not only rapid empirical therapy institution but also a rapid de-escalation [23]. Although the high cost may pose a potential barrier to their use, their utilization should be encouraged to enable the initiation of effective therapy as quickly as possible, especially in cases of severe clinical infection, as in the case of high suspicion of MDRO infection [24].

### Therapeutic options

A multidisciplinary management is of paramount importance for preventing the spread of resistant rods and ensuring a timely, effective treatment [4, 25]. Clinical history and the use of tools to predict the risk of MDRO infection should be employed when selecting antibiotic therapy. In recent years, infection severity has guided the choice of empirical antibiotic therapy, typically consisting of two antibiotics from different classes [6, 8, 23].

When an MDRO infection is highly suspected, and when the clinical presentation is severe (e.g., septic shock) the use of appropriate large-spectrum therapies, involving new molecules such as ceftazidime/avibactam, cefiderocol, daptomycin is justified. It should be followed by a rapid de-escalation, if possible, once microbiological tests turned out positive, to reduce resistance rates. An appropriate administration of such antibiotics—such as continuous infusion in the case of beta-lactams [26]. – appears of utmost importance, too, to increase their effectiveness, especially in sickest patients with ascites who have a high volume of distribution.

The management of MDRO infections, especially if severe, also relies on non-antibiotic therapies, which are similar to what is commonly applied in sepsis. These strategies include adequate caloric support, appropriate resuscitation strategies, management of vascular catheters responsible for the infection, and source control [27].

### Outcome

The outcome of patients with ESLD, especially with ACLF, appears to be significantly reduced when a superimposed infection occurs. For instance, a study by Fernandez and Colleagues demonstrated that bacterial infection significantly increased mortality in patients with ACLF (90-d survival 49% vs 72%), being these independently associated with mortality, both in patients with ACLF-1 and ACLF-2 [28]. The prognosis seems to be even worse when the infection is caused by MDRO strains. Indeed, the aforementioned multicenter study from Europe confirmed that these infections were associated with a lower resolution rate (71.4% *vs.* 87.6%), a higher prevalence of severe sepsis/shock (31.9% *vs.* 12.2%,), ACLF (67.5% *vs.* 45.6%) and, ultimately, a significantly higher 28-day mortality (35.1% *vs.* 18.1%) [13]. Taken together, these data pose many questions regarding sickest patients with ACLF awaiting LT and experiencing MDRO infection. On one hand, the bad short-term prognosis in terms of transplant-free survival, counterbalanced by the good post-operative survival in patients with ACLF [29], could represent a factor to proceed with the transplant. Conversely, a severe, uncontrolled MDRO infection with multiorgan failures should be viewed as a condition where the transplantation may be potentially inappropriate.

Therefore, the decision to proceed with transplantation in patients with MDRO-controlled infection should be made on a case-by-case basis, identifying the correct **timing** and balancing the benefits of transplantation with the risks associated with the type of infection (pandrug, extended drug resistance), source control, and available therapeutic options for the post-operative phase [23, 30, 31].

### Prevention

Preventing and reducing modifiable risk factors for MDRO infection in ESLD not only represents a research field, but it will be also a major goal for the future. A continuous update of local epidemiology (indeed, many studies have documented significant changes in the prevalent MDR strain within the same ward) and the stratification of patients at higher risk, based on the available tools, may be helpful and cost-effective preventive strategies.

A further option is to foster antibiotic stewardship, which appears essential for reducing antibiotic pressure, and consequently, the risk of colonization and infection from MDR organisms [4].

Since rectal colonization confers a high risk of subsequent invasive infection, fecal microbiota transplantation could be promising preventive strategy in pre-transplant candidates. Bajaj et al. summarized the current evidence regarding this option in many settings of cirrhosis, with favorable results [32]. There are, however, major points to be considered. First, indications are heterogeneous (alcoholic hepatitis, Cl. Difficile infection, hepatic encephalopathy) and, at present, do not specifically include resolution of colonization. Moreover, the role of microbiota transplantation as a game changer in liver disease progression, through an improvement of inflammation, warrants investigations. Second, it is currently unknown what is the preferable method (capsules, enemas, colonoscopy). Third, there are safety issues regarding donor screening and the risk of intestinal infection.

Finally, infection prevention requires a series of "non-pharmacological" strategies that are markedly beneficial for patients, such as the proper management of devices (e.g., venous and arterial vascular catheters, ventilators), as well as hand hygiene and the use of safety devices. We believe that training new operators and retraining experienced ones, along with evaluating the trends of MDRO infections and continuously updating protocols, can be valid options.

## MDRO infection after LT

### Prevalence

The prevalence of BI in the early phase after LT ranges from 30 to 60%, being surgical site infections, catheter-related bloodstream infections, and ventilator-associated pneumonia the commonest sources. Considering MDRO infections gram-negative rods are the most frequently encountered, presenting mostly as intrabdominal infection or nosocomial pneumonia. ESBLE infections (mainly K. pneumoniae and E. coli) range from 5.5% to 7%, whereas CRE infections range from 6% to 12.9% [33]. A rising prevalence of gram-positive infection has been encountered, too, as showed in a recently published paper from Spain, where *E. Faecium* was the commonest resistant strain [34].

### Risk factors

There are three main scenarios for the development of post-LT MDRO infection.

First, **donor-derived infections (DDI) caused by MDRO**. Deceased donors are critically ill patients admitted to the ICU and have several risk factors for developing or acquiring MDRO colonization or infection. Lewis et al. [35] summarized the available literature in this field, showing that around 50% of solid transplant recipients who received a graft from a donor with MDRO infection, subsequently acquired a DDI. Another study reported 15% prevalence of MDRO-related infections among 440 solid organ donors, with Hepatitis C, dialysis, prior hematopoietic cell transplant, and exposure to antibiotics with a narrow Gram-negative spectrum acting as independent predictors [36]. A further study carried out in Italy on 759 deceased donors reported 36 cases of CRE infection, with culture positivization occurring after transplantation in most cases: when the transplant was not related to the site of CRE infection, 90-d post-transplant mortality was low (8.3%). Conversely, when transplants were performed in the presence of CRE in the same site and/or in blood, mortality was higher (23.1%); in two cases (one lung and one liver), death was associated with early post-transplant CRE sepsis due to the same CRE species as found in the donor [37]. Another US study showed that 22 out of 182 patients who underwent LT in 2015 and 2016 received a MDRO-positive donor, with 3.8 higher odds to have a post-LT MDR infection [38]. Current Guidelines suggest that donors with MDRO infection require careful discussion with the Transplant Team and the Infectious Disease Specialist prior to accepting the graft, to weigh risk and benefits in a case-by-case basis [39].

Second, the development of an **MDR infection in a previously (pre-operatively) MDRO colonized and/or infected patients**. Strong data are available about the role of pre-LT colonization on the post-operative outcome, whereas the outcome of patients who acquired both pre- and post-LT MDRO infection is more difficult to ascertain. Indeed, the high mortality from a systemic MDRO infection in the sickest patients with cirrhosis and ESLD may often reduce the odds of being transplanted. As an example, a study on the Eurotransplant database by Friedrich et al. [40] showed that twenty-three LT candidates with pre-LT MDRO infection had significantly higher chances of being delisted than patients without infection, since the occurrence of MDRO infection led to a two-fold mortality risk while awaiting transplantation. A recent study from Brazil showed that among a large cohort of LT recipients, the development of pre-LT MDRO infection was an independent predictor of 90-d post-operative survival, and that the chance to develop MDRO infection post-operatively was higher in those experiencing the same infection before surgery. This represents a relevant point on a clinical ground because it helps to refine post-operative prognosis. It should be mentioned, however, that patients who experienced a pre-LT MDRO infection were sickest (e.g., ACLF prevalence 62% vs 7%, median MELD at transplant 30 vs 20), therefore transplant may be viewed as the best available therapeutic option [41]. A recently published multicenter, retrospective study from Spain showed that MDRO infection were found in 21.7% of cases after transplantation. Again, a previous MDR infection occurring within 3 months before LT, was identified as an independent predictor of such infections, together with a number of packed red blood cells and prior ICU admission [34].

Pre-LT colonization has been considered a risk factor for post-LT MDRO colonization and subsequent infection. Robust data are now available on CRE strains. Giannella et al. [10] demonstrated in a prospective cohort of 237 LT patients that CRE colonization before LT was an independent risk factor for post-LT CRE infection. The same group [42] evaluating the occurrence of CRE systemic infections in a larger cohort of 553 LT recipients, demonstrated that CRE colonization at LT was an independent risk factor for the occurrence of the same infection within one year after LT, along with post-LT colonization, combined transplant, prolonged mechanical ventilation, re-intervention, and rejection. These data were confirmed by another prospective study from Brazil [43] and by a multinational multicenter study that proposed an easy-to-use tool to predict post-operative MDR infection [11]. Regarding carbapenem-resistant *Acinetobacter baumannii,* it has been confirmed that patients who experienced such an infection were more prone to colonization before surgery [44].

Robust data are also available for gram-positive MDR pathogens. A meta-analysis of twenty-three studies on solid organ transplantations (seventeen on LT) [45] confirmed that pre-transplant and post-transplant methicillin-resistant staphylococcal colonization increased the risk of post-transplant systemic infection (pooled risk ratio: 5.51; 95% CI 2.36–12.90 and 10.56; 95% CI 5.58–19.95, respectively); similarly, pre-transplant and post-transplant VRE colonization was also associated with a significant risk of VRE infection (relative risk 6.65; 95% CI 2.54–17.41 and 7.93; 95% CI 2.36–26.67, respectively).

The last scenario is the **occurrence of MDRO infection in a previously uninfected, uncolonized patient**. Risk factors for this type of infection are often related to post-operative complications (both graft-related and unrelated). These may increase antibiotic pressure, prolong mechanical ventilation, and the ICU length of stay.

### Outcome

BI occurrence in the early postoperative days often leads to impairment of graft function, and of extrahepatic organ(s), according to the source of infection; as a consequence, this leads to a prolonged ICU stay, need for immunosuppression adjustment (with increased risk of rejection). The occurrence of an MDRO infection has been also associated with an even more worsened post-LT outcome: mortality after these infections has been shown to be up to five times higher than that observed for non-MDRO infections [33, 46, 47]. The mortality rate after MDR infection occurring in the early post-operative phase is quite variable among studies, even after stratifying the outcome based on the dominant strain. According to a literature review conducted by Bartoletti et al., infections by carbapenem-resistant Acinetobacter baumannii and Pseudomonas Aeruginosa carried a 60-d post-LT mortality up to 40%, whereas MRSA infection carried a 25% mortality within 30 days [48].

### Therapeutic strategies

In patients who have recently overcome an MDRO bacterial infection before surgery, a careful evaluation of targeted pre-transplant antibiotic prophylaxis seems appropriate. Although the issue of intraoperative antibiotic prophylaxis is debated and inconsistently applied [49], a recent multicenter study showed the application of targeted antibiotic prophylaxis up to 84% of cases in LT candidates with previous gram-negative MDR infection [50].

Additionally, appropriate management of immunosuppression, with the possibility of using drug combinations and/or IL2-RA induction to minimize or delay standard immunosuppressive therapy, appears to be a helpful therapeutic strategies, as typically recommended for critically ill patients. Notably, induction therapy is not associated with a higher risk of post-transplant infection [51].

Recently published guidelines by the American Society of Transplantation Infectious Diseases suggest the use of novel molecules, stratifying the patients according to multi-resistant rods. In detail, ceftazidime/avibactam and meropenem/vaborbactam should be considered first-line treatments for CRE infections. High-dose continuous or extended-infusion antipseudomonal β-lactams, ceftolozane/tazobactam, or ceftazidime/avibactam may be used for multi- or extended-drug resistant Pseudomonas aeruginosa. Finally, carbapenem and polymyxin combination therapy can be used as the treatment of choice for Acinetobacter baumannii [52].

**Fig. 1:**
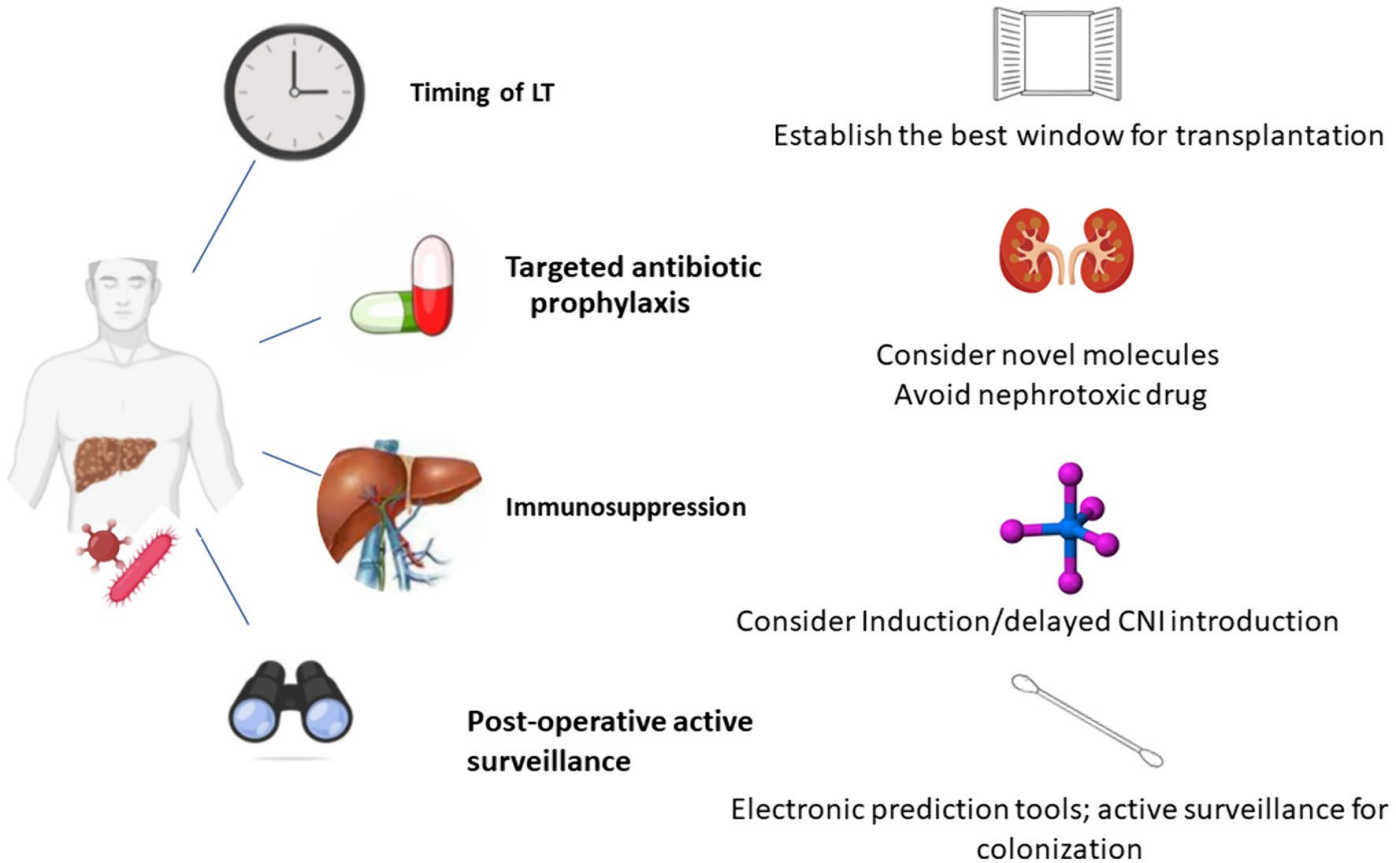
Issues in liver transplantation for patients with recent severe MDRO infection

Regarding antimicrobials, a careful evaluation regarding colistin and aminoglycosides should be reserved in the post-transplant setting, balancing their effectiveness against multidrug-resistant rods with the risk of nephrotoxicity. Moreover, a close consultation with Infectious Disease Specialists, to predict the risk of MDRO infection with available tools, to institute adequate antimicrobial therapy and to enable de-escalation, appears of paramount importance (Fig. 1). Finally, it’s worth noting that the antibiotic pressure to which such patients may be exposed can lead to a higher risk of invasive fungal infection, thus considering dedicated prophylaxis may be useful.

### Prevention

The non-antibiotic measures previously described in the context of ESLD (e.g., hand hygiene, microbiological surveillance) are even more relevant in the post-operative and intensive care settings, which are exposed to high antibiotic pressure. In addition to these measures, selective digestive decontamination has been previously proposed as a therapeutic strategy, for instance in colonized patients. A meta-analysis of four randomized studies published between 1994 and 2002 by Safdar et al. showed that it reduced the incidence of gram-negative infections (84% reduction), whereas it had no effect on overall infections [53]. These results have been confirmed in a further meta-analysis by Gurusamy et al., who did not find significant differences in mortality or post-LT infections after comparing this strategy with placebo, prebiotics, and probiotics [54]. Therefore, adding these disappointing results to the actual risk of drug resistance [55], current Guidelines do not advise towards this preventive option [52]. Selective intestinal decontamination may have a role in the setting of a living donor LT, where surgery could be more effectively planned. However, a prospective study using oral colistin plus gentamicin three days before surgery until the day of discharge did not find a decrease in the post-LT infection rate (42.7% vs. 46.8%) [56].

## Conclusions

Bacterial infections still represent a significant complication for patients with ESLD and in the immediate post-LT phase. MDRO infection further worsens the prognosis, both in the pre- and post-operative periods (Table 1). For this reason, proper surveillance, prompt diagnosis, and correct treatment are of significant importance in improving patient survival. We believe it is appropriate to consider the impact of MDR bacterial infection in ESLD patients in a different, more focused way, if the patient is a candidate for LT, by implementing a more aggressive management of risk factors and targeted prophylaxis at the time of transplantation. On the other hand, the management of an infection in the immediate post-liver transplant period cannot be properly treated without a thorough evaluation of the patient’s medical history, including the presence of risk factors for the development of MDR infections.

**Table 1:** The MDRO scenario in ESLD awaiting liver transplantation and in liver transplant recipients

	ESLD patients, awaiting liver transplantation	Liver transplant recipients, early post-operative phase
Prevalence of MDRO infections	20–30% of culture-positive bacterial infections	5–20% of culture-positive bacterial infections
	Marked differences in prevalence and type of MDR strains among countries and centres	Marked differences in prevalence and the type of MDROs among countries and centres
Risk Factors for MDRO infections	Previous bacterial infection(s) (especially if MDRO)	Donor-derived infection caused by MDRO
	MDRO colonization	MDRO colonization before and/or after LT
	Recent and/or ongoing long-term antibiotic therapie	Recent and/or ongoing long-term antibiotic therapies
	Indwelling catheters	Indwelling catheters, prolonged mechanical ventilation
	Invasive procedures	Invasive procedures (including re-operation)
	Prophylaxis*	Microbiological samples with detailed report of mechanisms of antibiotic resistance
Tools for diagnosis of MDRO infections	Microbiological samples with detailed report of mechanisms of antibiotic resistance	Use of novel diagnostic kits able to shorten turnaround times
	Use of new diagnostic kits able to shorten turnaround times	
Novel therapies for MDRO infection	New molecules to be used for empirical/targeted antibiotic therapy	New molecules to be used for empirical/targeted antibiotic therapy
Specific issues in Liver Transplant setting	Consider appropriate window and timing for LT, balancing benefit and futility of transplant	Patient and graft survival is significantly impaired in patients with MDRO infection than in patients with non-MDRO infection or without any infection
	Uncontrolled MDRO infection may be viewed, especially in ACLF patients, as a contraindication to transplantation	
Preventive strategies	Hand hygiene	Hand hygiene
	Antibiotic stewardship	Antibiotic stewardship
	Update of local epidemiology (especially in ICU)	Update of local epidemiology (especially in ICU)
	Fecal microbiota transplantation**	Targeted antibiotic prophylaxis at the time of LT^§^
		Adoption of targeted immunosuppressive strategies in high-risk patients
		Selective digestive tract decontamination**

*ESLD* end-stage liver disease, *ICU* intensive care unit, *LT* liver transplantation, *MDRO* multidrug resistant organism. *Long-term antibiotic prophylaxis should be reserved only to patients with prior spontaneous bacterial peritonitis and variceal bleeding. **Debated indication, requiring more data. ^§^in those patients with prior MDRO infection, MDR colonization

## Data Availability

Not applicable.

## Abbreviations

ACLF: Acute-on-chronic liver failure
BI: Bacterial infection
CRE: Carbapenem-resistant enterobacterales
DDI: Donor-derived infection
ESBLE: Extended-spectrum beta-lactamase enterobacterales
ICU: Intensive care units
LT: Liver transplantation
MDRO: Multidrug-resistant organism
SDD: Selective digestive decontamination
VRE: Vancomycin-resistant enterococci
